# Feeling the weight of post‐COVID fatigue

**DOI:** 10.1113/EP094274

**Published:** 2026-09-10

**Authors:** Demetris S. Soteropoulos, Mark R. Baker

**Affiliations:** ^1^ Biosciences Institute Newcastle University Newcastle upon Tyne UK; ^2^ Translational and Clinical Research Institute Newcastle University Newcastle upon Tyne UK

**Keywords:** post‐COVID, processing, sensory

Understanding the pathophysiology of fatigue in long‐term medical conditions, where chronic fatigue is a major symptom, is an important first step towards developing targeted therapeutic interventions. Whilst fatigue is likely to be multi‐factorial, one testable mechanism posits that individuals experience fatigue because of the failure of the sensory system to judge the effort required for action. Consequently, movement is less efficient and perceived to require more effort, exacerbating fatigue. In this issue of *Experimental Physiology*, Thomas et al. (2026) show that there is no evidence of significant central sensory processing abnormalities in patients with post‐COVID fatigue (also referred to as long COVID fatigue or post‐COVID syndrome fatigue) compared to controls. The authors tested two phenomena involved in somatosensory processing (Kilteni & Ehrsson, 2022): sensory gating, which is the suppression of externally generated somatosensory input during movement relative to rest; and sensory attenuation, which refers to the reduced perception of predictable afferent feedback generated by movement. This result is in line with the only other published study investigating sensory gating in patients with post‐COVID fatigue (Baker et al., 2023) but contrasts with underlying mechanisms of fatigue in other conditions, including neurological disorders such as stroke (Kuppuswamy, 2025). Importantly, the study by Thomas et al. was conducted as part of *Experimental Physiology*’s Registered Research Report initiative (Rasmussen et al., 2025), with the study protocol, including the hypotheses, design and planned analyses, published in advance (Thomas et al., 2024). Whilst not necessarily appropriate for all discovery science (Rasmussen et al., 2026; Richter et al., 2026), for a study investigating pathophysiology in clinical populations this approach reinforces confidence in the conclusions.

In chronic fatigue, two contributory components are recognized; one is ‘trait fatigue’ and the other is ‘state fatigue’. Trait fatigue is the stable, longer‐term sense of being fatigued over weeks or months, whereas state fatigue is the moment‐to‐moment experience of fatigue, which can change during or after activities, such as completing a motor task during an experiment. Importantly, in this study, the authors (Thomas et al., 2026) showed that in the same patient group, both trait fatigue and state fatigue were elevated, yet with no significant reduction in cognitive or motor performance. Despite the absence of deficits in central sensory processing, the behaviour of this group was typical of patients with fatigue; other mechanisms that increase perceived exertion without affecting performance must therefore contribute. Primary muscle pathology (Baker et al., 2023; Germann et al., 2025), is one very plausible candidate that merits much closer scrutiny. Other mechanisms such as altered central motor drive, autonomic dysregulation, and the way bodily signals are interpreted and weighted (dysfunction or failure of interoception or ‘active inference’) may also contribute, whether independently or in combination. Experimental approaches that can disentangle which of these mechanisms, alone or in combination, underlies the dissociation between perceived effort and preserved performance will be important if the field is to progress.

Persistent post‐infection fatigue can be profoundly disabling. Since the emergence of the COVID‐19 pandemic, persistent post‐infection fatigue has become widespread and as a society we are now grappling with a post‐COVID fatigue epidemic of our own, affecting ∼2 million in the UK. Unpicking the pathophysiology of fatigue is therefore essential if we are to address this problem at a societal level. The work of Thomas et al. underscores just how important this task is and helps refine how we think about fatigue itself: the mechanisms driving fatigue after stroke need not be the same mechanisms driving fatigue as post‐COVID fatigue, even though both are lumped together as ‘fatigue’. Given such heterogeneity, sub‐phenotyping of the underlying pathophysiology of fatigue will be essential before we can progress to developing trial outcome measures that are specific to fatigue subtype, or rational, precision therapies for this common and debilitating symptom.

## AUTHOR CONTRIBUTIONS

Both authors have read and approved the final version of this manuscript and agree to be accountable for all aspects of the work in ensuring that questions related to the accuracy or integrity of any part of the work are appropriately investigated and resolved. All persons designated as authors qualify for authorship, and all those who qualify for authorship are listed.

## CONFLICT OF INTEREST

The authors declare they have no conflicts of interest.

## FUNDING INFORMATION

No funding was received for this work.

## GENERATIVE AI STATEMENT

No AI tool has been used in the preparation of this Viewpoint.
